# Effectiveness of early glucocorticoids in myasthenia gravis: a retrospective cohort study

**DOI:** 10.3389/fneur.2023.1259484

**Published:** 2023-12-19

**Authors:** Lulu Zhen, Xue Zhao, Wenbo Li, Jinru Wu, Haodong Shang, Shufan Chen, Xiaoyan Zhu, Yiren Wang, Xiaoxiao Yu, Guanlian Hu, Zhan Sun, Yingna Zhang, Jing Zhang, Hua Fang, Yunke Zhang, Qingyong Zhang, Xinzheng Cui, Jie Lv, Junhong Yang, Feng Gao

**Affiliations:** ^1^Department of Neurology, The Second Affiliated Hospital of Zhengzhou University, Zhengzhou, China; ^2^Department of Neuroimmunology, Henan Institute of Medical and Pharmaceutical Sciences, Zhengzhou University, Zhengzhou, China; ^3^Basic Medical College, Zhengzhou University, Zhengzhou, China; ^4^BGI College, Zhengzhou University, Zhengzhou, China; ^5^Department of Encephalopathy, First Affiliated Hospital of Henan University of Chinese Medicine, Zhengzhou, China; ^6^Myasthenia Gravis Comprehensive Diagnosis and Treatment Center, Henan Provincial People’s Hospital, Zhengzhou, China

**Keywords:** myasthenia gravis, glucocorticoids, early treatment with GC, delayed treatment with GC, treatment target, prognostic factors

## Abstract

**Purpose:**

This study aimed to clarify the effect of early glucocorticoid (GC) application on achieving minimal manifestation (MM) status or better in the treatment of myasthenia gravis (MG) in the early clinical phase.

**Methods:**

A retrospective analysis was performed using data from 336 patients with MG who received GC therapy from January 2015 to September 2022 in the Zhengzhou University Henan Institute of Medical and Pharmaceutical Sciences Myasthenia Gravis Biobank (ZMB). Patients were divided into two groups: the early mono-GC group (treated with GC within 6 months of MG onset) and the delayed mono-GC group.

**Results:**

Kaplan–Meier analysis showed that the early mono-GC group achieved MM status earlier and more frequently than the delayed mono-GC group (log-rank test, *p* = 0.0082; hazard ratio [HR], 1.66; *p* = 0.011). The early mono-GC group had a lower maintenance oral GC dose than the delayed mono-GC group. In multivariate Cox regression analysis, early mono-GC (HR, 1.50; *p* = 0.043), early-onset MG (EOMG) (HR, 1.74; *p* = 0.034), and ocular MG (OMG) (HR, 1.90; *p* = 0.007) were associated with MM status or better. In conclusion, early mono-GC, EOMG, and OMG were positive predictors of treatment goals. In EOMG, OMG, and acetylcholine receptor antibody-positive MG (AChR-MG) subgroups, the maintenance oral GC doses in the early mono-GC group were significantly lower than the doses in the delayed mono-GC group (*p* < 0.05).

**Conclusion:**

Early intervention with GC led to better long-term outcomes and reduced the necessary maintenance dose of oral GC for patients with MG. EOMG and OMG were positive predictors of MM status or better with mono-GC.

## 1 Introduction

Myasthenia gravis (MG) is an acquired autoimmune disease predominantly caused by damage to the acetylcholine receptor (AChR) on the postsynaptic membrane of the neuromuscular junction (NMJ). It is clinically characterized by skeletal muscle weakness and easy fatigue (1, 2). GC is the first-line agent of choice for MG because of its rapid onset, low cost, and high efficiency (3–6).

However, the clinical use of GC treatment is still hampered by uncertainties surrounding whether and when to take it. Several studies have shown that when GC is taken within 1 year of MG onset, remission may be induced, but the relationship between GC administration within 6 months and earlier remission has not been studied. Pascuzzi et al. (6) suggested that GC administration within the first 1.3 years of the course of MG can induce remission, while administration after 4.4 years is unlikely to induce remission. Tarin et al. (7) conducted a study in 87 patients with MG and steroid-treated persistent ophthalmoplegia and/or ptosis, which revealed that patients who started treatment within 12 months of onset (early treatment group), compared with those who started treatment 12 months later (delayed treatment group), had a 2-fold chance of complete remission of ophthalmoplegia. Other retrospective studies have provided evidence that early GC use in patients with ocular MG (OMG) may delay or reduce the risk of potential symptom generalization and worsening (8–10). In addition, to date, no guideline distinguishes whether to simultaneously take GC and pyridostigmine at the beginning of disease progression or when the symptoms improve (11, 12). Furthermore, patients often refuse GC treatment due to its side effects, and approximately 20–40% of patients with MG develop worsening symptoms, increasing the risk of relapse with prolonged disease (13–15). The above studies did not clearly define the benefits of GC use at 0–6 months, and clinical data are lacking on whether earlier remission may be achieved and recurrence may be reduced under these circumstances. Therefore, further exploration of the timing of GC treatment and the impact of early GC intervention on achieving MG treatment goals is necessary.

Therefore, we conducted a retrospective cohort analysis of patients with MG treated with GC at the ZMB to clarify the impact of GC administration within 6 months on achieving MG treatment goals and to provide insight and guidance for the early clinical application of GC.

## 2 Materials and methods

### 2.1 Patients

The medical records and follow-up data of patients with MG in the Zhengzhou University Henan Institute of Medical and Pharmaceutical Sciences Myasthenia Gravis Biobank (ZMB) from January 2015 to September 2022 were retrospectively analyzed. On-site, video, or telephone follow-ups were performed for all enrolled patients and were completed in December 2022.

The inclusion criteria were as follows: (a) The diagnostic criteria for MG are as follows (1) (i.e., [i] fluctuating muscle weakness, worsened by exertion and improved by rest; [ii] positive neostigmine test; [iii] serum-positive AChR or muscle-specific tyrosine kinase antibody (MuSK) antibodies; and [iv] positive repetitive nerve stimulation (RNS): The amplitude of the 4th or 5th compound muscle action potential decreases by >10% from the first amplitude or by >30% with high-frequency stimulation). On the basis of satisfying [i], MG is diagnosed if any of [ii], [iii], or [iv] are satisfied; (b) Age of onset of 1–80 years; (c) Included patients with MG have been treated with GC for at least 1 month.

The exclusion criteria were as follows: (a) MG diagnosis at ages of <1 year or > 80 years; (b) presence of other systemic tumors such as respiratory, digestive, urinary, and hematologic tumors (e.g., lung cancer, pancreatic cancer, gastric cancer, bladder cancer, kidney cancer, lymphoma, leukemia, etc.). Thymoma is excluded; (c) prognosis assessment of the impact of mental illness; (d) patients with other concomitant diseases that seriously jeopardize the safety of patients; or accompanied by serious underlying diseases, such as liver, kidney failure, heart failure, etc.; (e) use of other immunosuppressants or targeted drugs before taking GC; (f) experience with GC therapy due to other diseases and presence of any contraindications to GC treatment; and (g) received GC treatment for less than 1 month.

### 2.2 Methods

#### 2.2.1 Data collection and research subgroups

Clinical features were collected, including the age of onset; sex; involved muscle group; level of autoantibodies; diagnostic tests; thymic status; initial, maximal, and maintenance GC doses; clinical severity using the Myasthenia Gravis Foundation of America (MGFA) (16) clinical classification at the time of onset and when the disease was most severe; and record the time from the beginning of GC treatment to the time that minimal manifestation (MM) was first reached; the number of patients who achieved MM status or better at the last follow-up; incidence of relapse and crisis; and GC-related adverse effects.

All early and delayed groups in our study were defined with a cut-off time of 6 months. The patients in our study were divided into two groups based on whether they took other non-steroidal immunosuppressants: the mono-GC group (no other non-steroidal immunosuppressants) and the combination-therapy group. OMG was defined as isolated the muscles of the eyes and eyelids (levator palpebrae superioris) involvement for a duration of >24 months (17, 18). Muscle group weakness other than the extraocular muscles was defined as generalized MG (GMG). EOMG referred to MG with a first onset before the age of 50 years, and late-onset MG (LOMG) referred to a first onset after the age of 50 years. When AChR but not MuSK antibodies were detected in serum, MG was called AChR-MG. Conversely, when MuSK but not AChR antibodies were detected in serum, MG was called MuSK-MG. Cases in which serological tests did not detect antibodies to AChR or MuSK were described as serologically-negative MG (SNMG). Titin antibody positive MG (Titin-MG) is titin antibody positive in serum, and LRP4 antibody positive MG (LRP4-MG) is LRP4 antibody positive in serum. Maximum oral GC dose: The maximum GC dose in our study was the highest dose of oral GC in a single day during treatment. Maintenance oral GC dose: a single day oral lower dose after symptoms of muscle weakness are controlled. Thymus type was determined by chest computed tomography (CT) and postoperative pathology. Diagnosis delay was defined as the difference between the date of diagnosis and the date of disease onset, and the follow-up time (disease duration) was defined as the interval between the diagnosis and the last date of follow-up or death. Relapse: When a patient whose original symptoms have disappeared or subsided relapse or worsen after a period of time.

#### 2.2.2 Clinical outcome assessment

The clinical severity at onset and at the most severe stage was assessed according to the MGFA clinical classification. Clinical outcomes and responses to treatment were assessed according to MGFA-post intervention status (MGFA-PIS), and the treatment goals for MG were MM, pharmacological remission (PR), and complete stable response (CSR), defined as MM status or better status (16). where MM: asymptomatic or mild symptoms, most of the time no impact on life, fatigue test can not reach normal (some patients have mild symptoms but fatigue test is normal is also listed in MM), with or without immunotherapy and pyridoxamine bromide, and then divided into MM-0, MM-1, MM-2 and MM-3 according to the definition of MGFA-PIS, a total of 4 subcategories. PR/CSR: asymptomatic, no impact on life, normal fatigue test, with or without immunotherapy, but not pyridigine bromide.

The primary outcomes were comparisons of the times for initiation of GC to first MM achievement and cumulative probabilities of MM achievement in the early and delayed mono-GC groups. The secondary outcomes were comparisons of oral GC doses, recurrence, crisis, and incidence of adverse effects between the two groups.

#### 2.2.3 Serological testing

The RSR Limited enzyme-linked immunosorbent assay (ELISA) kit (RSR Limited, UK) was used to detect AChR antibodies, and the DLD Diagnostika ELISA kit (DLD Diagnostika, Hamburg, Germany) was used to detect Titin antibodies using the cut-off values of 0.45 nmol/L and 1, respectively (19, 20).

MuSK antibodies and LRP4 antibodies were detected by a cell-based assay (CBA), as previously described (20, 21). Briefly, HEK293 cells were transfected with the corresponding plasmids, causing green fluorescence, and the secondary Alexa Fluor TM568-labeled sheep anti-human IgG antibody (Thermo Fisher Scientific) caused red fluorescence. Two researchers independently observed the cells under a two-color fluorescence microscope. The fluorescence intensity was judged by the number of fluorescent cells.

#### 2.2.4 Statistical analysis

Quantitative data that did not conform to the normal distribution are presented as median and interquartile ranges (IQR). For categorical variables, the data are presented as numbers (percentages). The chi-square (*χ^2^*) test and Fisher’s exact test were used to compare categorical variables, and the Mann–Whitney *U* test or Kruskal–Wallis test was used to compare quantitative variable differences. The Kaplan–Meier method and log-rank test were used to analyze the time to reach MM after starting GC treatment. Cox regression analysis was used to estimate the HR and 95% confidence intervals (CIs) of treatment along with other relevant factors for achieving treatment goals. Using the IBM SPSS 26 statistical software for Windows (IBM Corp, Armonk, NY, USA) for data analysis, *p* < 0.05 indicated a statistically significant difference.

#### 2.2.5 Standard protocol approvals

All clinical investigations were conducted in accordance with the principles of the Helsinki Declaration. This study was approved by the Medical Ethics Committee of Henan Medical Science Research Institute of Zhengzhou University (YLL-002), and the patients signed informed consent forms.

## 3 Results

### 3.1 Demographic characteristics

**Figure 1:**
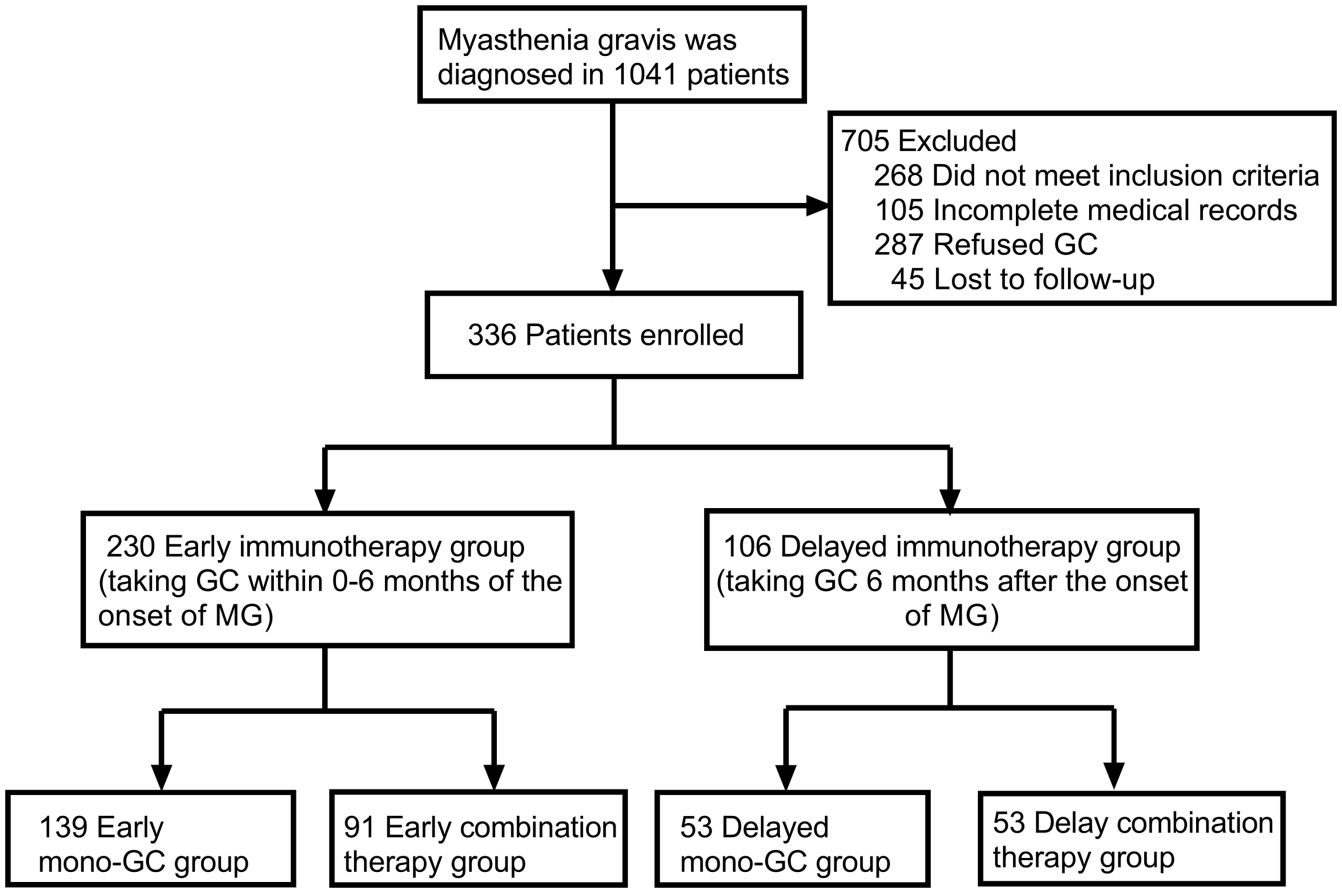
Flowchart shows patients included in the study.

From January 2015 to September 2022, a total of 1,041 patients were registered in the ZMB, and 336 patients with MG who had received immunotherapy were included in the study. Based on the time from onset to GC treatment, 230 patients were divided into the early immunotherapy group, of which 139 patients were in the early mono-GC group and 91 in the early combination-therapy group. Additionally, 106 patients were in the delayed immunotherapy group, of which 53 patients were in the delayed mono-GC group and 53 in the delayed combination-therapy group (Figure 1). The patients in the study included 192 women (57.1%) and 144 men (42.9%), with a male-to-female ratio of 1:1.33 and a median onset age of 36.5 years (IQR, 10.25–52.75 years). The patients in the early immunotherapy group had less involvement of the limb (*p* = 0.038) and bulbar muscle (*p* = 0.010) than the patients in the delayed immunotherapy group (Table 1). The patients for the early mono-GC group had less involvement of the cervical muscle (*p* = 0.006) than the delayed mono-GC group (Table 2).

**Table 1:** Baseline demographic and clinical characteristics in Early and Delayed immunotherapy group.

	Total (*N* = 336)	Early immunotherapy group (*N* = 230)	Delayed immunotherapy group (*N* = 106)	*p-*value
Age at onset, years, median (IQR)	36.5 [10.25, 52.75]	34.5 [6, 52]	39.5 [23.75, 53]	0.087^†^
Sex (female), *n* (%)	192 (57.1)	139 (60.4)	53 (50.0)	0.072
Disease duration, months, median (IQR)	52 [38, 79]	49.5 [37, 77]	55 [39, 84]	0.078^†^
Diagnostic delay, months, median (IQR)	1 [1, 2]	1 [1, 2]	1 [1, 3]	0.066^†^
Starting position, *n* (%)				
Ocular	262 (78.0)	182 (79.1)	80 (75.5)	0.452
Limb	33 (9.8)	23 (10.0)	10 (9.4)	0.871
Bulbar	44 (13.1)	28 (12.2)	16 (15.1)	0.461
Affected muscle group, *n* (%)				
Ocular	306 (91.1)	210 (91.3)	96 (90.6)	0.825
Limb	116 (34.5)	71 (30.9)	45 (42.5)	**0.038**
Bulbar	125 (37.2)	75 (32.6)	50 (47.2)	**0.010**
Cervical	22 (6.5)	11 (4.8)	11 (10.4)	0.054
Respiratory	39 (11.6)	23 (10.0)	16 (15.1)	0.175
Auto-antibodies, *n* (%)				
AChR	286 (85.1)	192 (83.5)	94 (88.7)	0.213
MuSK	6 (1.8)	4 (1.7)	2 (1.9)	1.000^*^
SNMG	38 (11.3)	30 (13.0)	8 (7.5)	0.139
AChR + MuSK	5 (1.5)	3 (1.3)	2 (1.9)	0.652^*^
Titin	70 (20.8)	49 (21.3)	21 (19.8)	0.754
LRP4	2 (0.6)	2 (0.9)	0	1.000^*^
Auxiliary examination				
Neostigmine test, n/N (%)	265/311 (85.2)	180/210 (85.7)	85/101 (84.2)	0.717
RNS, n/N (%)	107/227 (47.1)	68/146 (46.6)	39/81 (48.1)	0.820
Thymoma, n/N (%)	72/327 (22.0)	45/222 (20.3)	27/105 (25.7)	0.267

AChR, acetylcholine receptor; MuSK, muscle-specific tyrosine kinase; SNMG, seronegative myasthenia gravis; RNS, repetitive nerve stimulation; IQR, interquartile range. ^*^Fisher’s exact test ^†^Mann–Whitney *U*. Values in bold indicate a statistically significant difference between the two groups.

**Table 2:** Baseline demographic and clinical characteristics in Early and Delayed mono-GC group, Early and Delayed combination therapy group.

	Early mono-GC group (*N* = 139)	Delayed mono-GC group (*N* = 53)	*P-*value	Early combination therapy group (*N* = 91)	Delayed combination therapy group (*N* = 53)	*P-*value
Age at onset, median (IQR)	24 [5, 50]	34 [16, 51.5]	0.210^†^	44 [24, 54]	42 [30.5, 53]	0.525^†^
Sex (female), *n* (%)	85 (61.2)	28 (52.8)	0.295	54 (59.3)	25 (47.2)	0.157
Disease duration, months, median (IQR)	52 [38, 80]	60 [42, 85]	0.234^†^	48 [36, 71]	53 [38, 84]	0.148^†^
Diagnostic delay, months, median (IQR)	1 [1, 2]	1 [0.5, 2]	0.641^†^	1 [1, 3]	2 [1, 4]	0.070^†^
Starting position, *n* (%)						
Ocular	115 (82.7)	41 (77.4)	0.394	67 (73.6)	39 (73.6)	0.996
Limb	13 (9.4)	3 (5.7)	0.408	10 (11.0)	7 (13.2)	0.691
Bulbar	14 (10.1)	9 (17.0)	0.188	14 (15.4)	7 (13.2)	0.721
Affected muscle group, *n* (%)						
Ocular	129 (92.8)	46 (86.8)	0.254^*^	81 (89.0)	50 (94.3)	0.373^*^
Limb	34 (24.5)	17 (32.1)	0.286	37 (40.7)	28 (52.8)	0.157
Bulbar	36 (25.9)	21 (39.6)	0.063	39 (42.9)	29 (54.7)	0.169
Cervical	2 (1.4)	6 (11.3)	**0.006** ^*^	9 (9.9)	5 (9.4)	0.929
Respiratory	8 (5.8)	7 (13.2)	0.128^*^	15 (16.5)	9 (17.0)	0.938
Auto-antibodies, *n* (%)						
AChR	112 (80.6)	45 (84.9)	0.487	80 (87.9)	49 (92.5)	0.390
MuSK	2 (1.4)	2 (3.8)	0.306^*^	2 (2.2)	0	0.532^*^
SNMG	21 (15.1)	4 (7.5)	0.164	9 (9.9)	4 (7.5)	0.768^*^
AChR + MuSK	3 (2.2)	2 (3.8)	0.617^*^	0	0	
Titin	23 (16.5)	6 (11.3)	0.366	26 (28.6)	15 (28.3)	0.972
LRP4	2 (1.4)	0	1.000^*^	0	0	--
Auxiliary examination						
Neostigmine test, n/N (%)	112/128 (87.5)	44/51 (86.3)	0.825	68/82 (82.9)	41/50 (82.0)	0.892
RNS, n/N (%)	42/87 (48.3)	19/38 (50.0)	0.859	26/59 (44.1)	20/43 (46.5)	0.807
Thymoma, n/N (%)	19/134 (14.2)	12/52 (23.1)	0.144	26/88 (29.5)	15/53 (28.3)	0.875

GC, glucocorticoid; AChR, acetylcholine receptor; MuSK, muscle-specific tyrosine kinase; SNMG, seronegative myasthenia gravis; RNS, repetitive nerve stimulation; IQR, interquartile range. ^*^Fisher’s exact test ^†^Mann–Whitney *U*. Values in bold indicate a statistically significant difference between the two groups.

### 3.2 Comparative analysis of early and delayed immunotherapy groups

**Figure 2:**
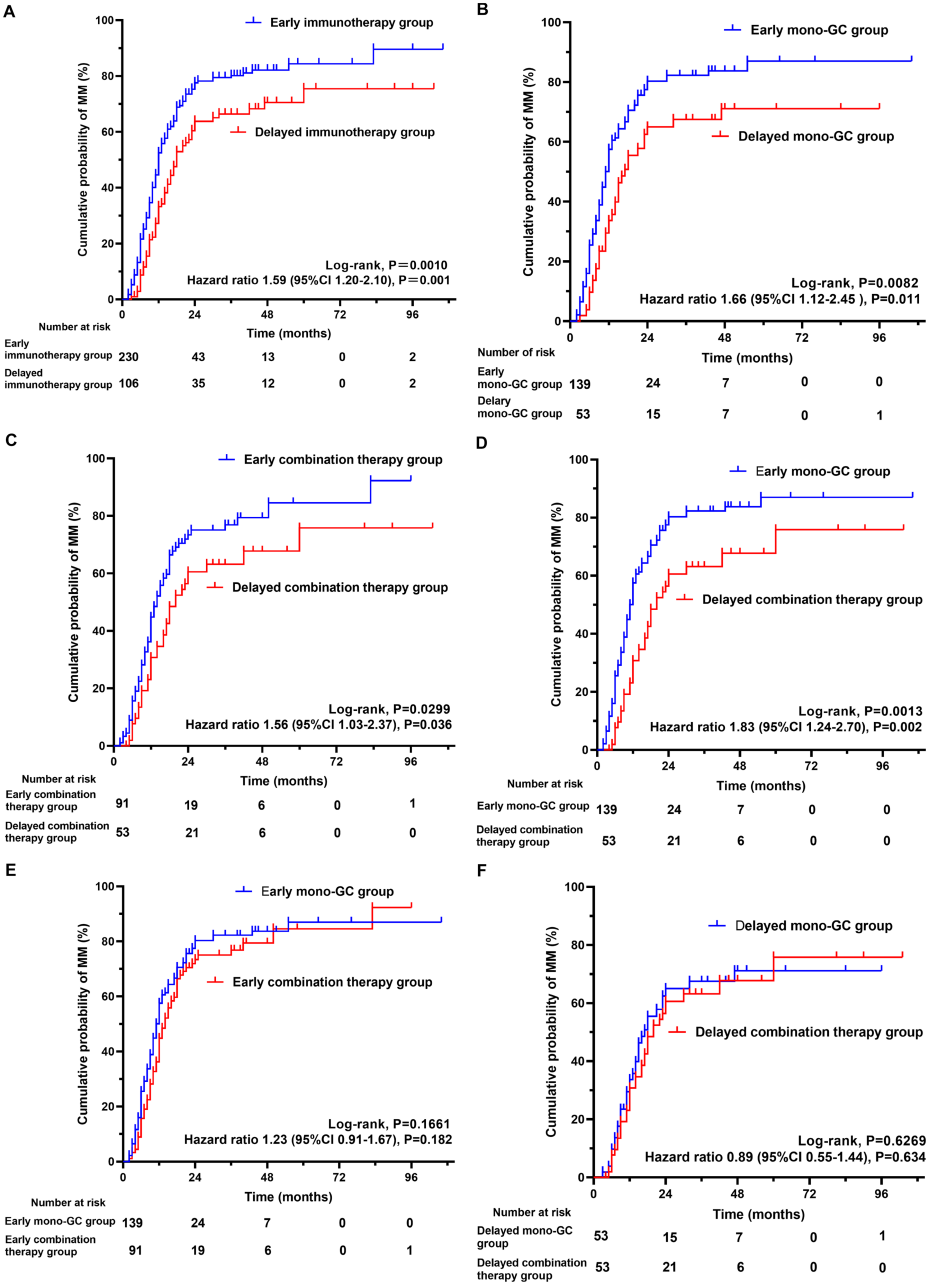
The cumulative probability of achieving minimal manifestation (MM) for myasthenia gravis. Kaplan–Meier curve showing the achievement of **(A)** MM (log-rank test, *P* = 0.0010; 95%CI 1.20–2.10, *P* = 0.001) in early immunotherapy group and delayed immunotherapy group; **(B)** MM (log-rank test, *P* = 0.0082; 95%CI 1.12–2.45, *P* = 0.011) in Early mono-GC group and Delayed mono-GC group; **(C)** MM (log-rank test, *P* = 0.0299; 95%CI 1.03–2.37, *P* = 0.036) in Early combination therapy group and Delayed combination therapy group; **(D)** MM (log-rank test, *P* = 0.0013; 95%CI 1.24–2.70, *P* = 0.002) in Early mono-GC group and Delayed combination therapy group; **(E)** MM (log-rank test, *P* = 0.1661; 95%CI 0.91–1.67, *P* = 0.182) in Early mono-GC group and Early combination therapy group; **(F)** MM (log-rank test, *P* = 0.6269; 95%CI 0.55–1.44, *P* = 0.634) in Delayed mono-GC group and Delayed combination therapy group; GC, glucocorticoid.

The Kaplan–Meier curve shows that the early immunotherapy group achieved MM earlier and more frequently than the delayed immunotherapy group (log-rank test, *p* = 0.0010). Univariate Cox regression analysis showed that the necessary HR and 95% CI to achieve MM were 1.59 and 1.20–2.10, respectively (*p* = 0.001) (Figure 2A). The time from GC administration to the first achievement of MM was significantly less in the early immunotherapy group than in the delayed immunotherapy group (10 [6, 15] vs. 12.5 [9,18], *p* = 0.004), with a MM status or better more often achieved at the final follow-up (60.0% vs. 48.1%, *p =* 0.041). The maximum and maintenance doses of oral GC were significantly lower in the early immunotherapy group than the doses in the delayed immunotherapy group (*p* < 0.05). No statistically significant differences were found in the incidences of recurrence, crisis, and thymotomy between the two groups (Table 3).

**Table 3:** Treatment and prognosis in Early and Delayed immunotherapy group.

	Total (*N* = 336)	Early immunotherapy group (*N* = 230)	Delayed immunotherapy group (*N* = 106)	*P-*value
MGFA classification at onset				0.836^*^
I, *n* (%)	258 (76.8)	178 (77.4)	80 (75.5)	
IIa, *n* (%)	34 (10.1)	24 (10.4)	10 (9.4)	
IIb, *n* (%)	37 (11.0)	24 (10.4)	13 (12.3)	
IIIb, *n* (%)	7 (2.1)	4 (1.7)	3 (2.8)	
MGFA classification at maximal worsening				0.106
I, *n* (%)	161 (47.9)	123 (53.5)	38 (35.8)	
IIa, *n* (%)	35 (10.4)	21 (9.1)	14 (13.2)	
IIb, *n* (%)	30 (8.9)	20 (8.7)	10 (9.4)	
IIIa, *n* (%)	22 (6.5)	13 (5.7)	9 (8.5)	
IIIb, *n* (%)	42 (12.5)	24 (10.4)	18 (17.0)	
IVa, *n* (%)	5 (1.5)	3 (1.3)	2 (1.9)	
IVb, *n* (%)	19 (5.7)	10 (4.3)	9 (8.5)	
V, *n* (%)	22 (6.5)	16 (7.0)	6 (5.7)	
Time from GC administration to MM, months, median (IQR)	11 [7, 11]	10 [6, 15]	12.5 [9,18]	**0.004** ^**†**^
MM or better status, throughout the course, *n* (%)	244 (72.6)	176 (76.5)	67 (63.2)	**0.011**
MM or better status, at last follow up, *n* (%)	189 (56.3)	138 (60.0)	51 (48.1)	**0.041**
Relapse, n/N (%)	190/322 (59.0)	125/221 (56.6)	65/101 (64.4)	0.187
Myasthenic crisis, *n* (%)	21 (6.3)	15 (6.5)	6 (5.7)	0.762
Initial oral GC dose, mg/day, median (IQR)	60 [30, 60]	60 [30, 60]	60 [30, 60]	0.176^†^
Maximal oral GC dose, mg/day, median (IQR)	60 [35, 60]	60 [30, 60]	60 [48.75, 60]	**0.017** ^**†**^
Maintain oral GC dose, mg/day, median (IQR)	5 [5, 10]	5 [5, 10]	10 [5, 15]	**0.001** ^**†**^
Pyridostigmine dose, mg/day, median (IQR)	180 [90, 180]	180 [90, 180]	180 [90, 240]	0.653
IVMP, *n* (%)	120 (35.7)	81 (35.2)	39 (36.8)	0.779
Tacrolimus, *n* (%)	79 (23.5)	49 (21.3)	30 (28.3)	0.160
AZA, *n* (%)	43 (12.8)	27 (11.7)	16 (15.1)	0.392
MMF, *n* (%)	16 (4.8)	13 (5.7)	3 (2.8)	0.259
Cyclosporine, *n* (%)	4 (1.2)	2 (0.9)	2 (1.9)	0.593^*^
Thymectomy, *n* (%)	87 (25.9)	53 (23.0)	34 (32.1)	0.079

MGFA, Myasthenia Gravis Foundation of America; MM, minimum manifestation; GC, glucocorticoid; AZA, azathioprine; MMF, mycophenolate mofetil; IQR, interquartile range. ^*^Fisher’s exact test. ^†^Mann–Whitney *U*. Values in bold indicate a statistically significant difference between the two groups.

The GC therapy caused one or more side effects in 205 (61.0%) of the 336 patients. The most common side effects were weight gain (37.6%) and Cushing-like appearance (26.7%). No statistically significant differences were observed in residual adverse effects (Table 4).

**Table 4:** Side effects and complications of glucocorticoid therapy.

	Total (*N* = 336)	Early immunotherapy group (*N* = 230)	Delayed immunotherapy group (*N* = 106)	*P*-value
Patients with any adverse events, *n* (%)	205 (61.0)	138 (60.0)	67 (63.2)	0.575
Total number of adverse events	255	173	82	
Cushingoid appearance	68	44	24	0.685
Weight gain	96	70	26	0.178
Cataracts	2	0	2	0.103^*^
Fundus changes	3	3	0	0.553^*^
Dyslipidemia	2	2	0	1.000^*^
Respiratory insufficiency	1	0	1	0.322^*^
Hypertension	19	13	6	1.000^*^
Diabetes	16	9	7	0.305
Osteoporosis	17	12	5	0.802
Necrosis of femur	3	2	1	1.000^*^
Bone and joint pain	2	2	0	1.000^*^
Infections	5	3	2	0.658^*^
Peptic ulcer	10	6	4	0.731^*^
Irritability	3	2	1	1.000^*^
Insomnia	3	2	1	1.000^*^
Skin rash/striae	5	3	2	0.658^*^

GC, glucocorticoid. ^*^Fisher’s exact test.

### 3.3 Comparative analysis of early and delayed mono-GC groups

The Kaplan–Meier curve shows that the early mono-GC group achieved MM earlier and more frequently than the delayed mono-GC group (log-rank test, *p* = 0.0082). Univariate Cox regression analysis revealed that the necessary HR and 95% CI to achieve MM were 1.66 and 1.12–2.45, respectively (*p* = 0.011) (Figure 2B). The time from GC administration to first achievement of MM was significantly less in the early mono-GC group than in the delayed mono-GC group (10 [6, 14.25] vs. 12 [8, 17.5], *p* = 0.039), with a MM status or better more often in the course of MG (79.1% vs. 62.3%, *p* = 0.017), and the maintenance oral GC doses were significantly lower than those in the delayed mono-GC group (*p* = 0.004). No statistically significant differences were identified in the incidences of recurrence, crisis, and thymotomy between the two groups (Table 5).

**Table 5:** Treatment and prognosis in Early and Delayed mono-GC group, Early and Delayed combination therapy group.

	Early mono-GC group (*N* = 139)	Delayed mono-GC group (*N* = 53)	*P-*value	Early combination therapy group (*N* = 91)	Delayed combination therapy group (*N* = 53)	*P-*value
MGFA classification at onset			0.498^*^			0.626^*^
I, *n* (%)	112 (80.6)	41 (77.4)		66 (72.5)	39 (73.6)	
IIa, *n* (%)	13 (9.4)	3 (5.7)		11 (12.1)	7 (13.2)	
IIb, *n* (%)	10 (7.2)	7 (13.2)		14 (15.4)	6 (11.3)	
IIIb, *n* (%)	4 (2.9)	2 (3.8)		0	1 (1.9)	
MGFA classification at maximal worsening			0.425^*^			0.204^*^
I, *n* (%)	90 (64.7)	28 (52.8)		33 (36.3)	10 (18.9)	
IIa, *n* (%)	11 (7.9)	3 (5.7)		10 (11.1)	11 (20.8)	
IIb, *n* (%)	8 (5.8)	4 (7.5)		12 (13.2)	6 (11.3)	
IIIa, *n* (%)	7 (5.0)	4 (7.5)		6 (6.6)	5 (9.4)	
IIIb, *n* (%)	14 (10.1)	7 (13.2)		10 (11.0)	11 (20.8)	
IVa, *n* (%)	0	1 (1.9)		3 (3.3)	1 (1.9)	
IVb, *n* (%)	4 (2.9)	4 (7.5)		6 (6.6)	5 (9.4)	
V, *n* (%)	5 (3.6)	2 (3.8)		11 (12.1)	4 (7.5)	
Time from GC administration to MM, months, median (IQR)	10 [6, 14.25]	12 [8, 17.5]	**0.039** ^**†**^	12 [7, 16.25]	14 [9, 20]	0.091
MM or better status, throughout the course, *n* (%)	110 (79.1)	33 (62.3)	**0.017**	66 (72.5)	34 (64.2)	0.293
MM or better status, at last follow up, *n* (%)	90 (64.7)	27 (50.9)	0.080	48 (52.7)	24 (45.3)	0.388
Relapse, n/N (%)	62/134 (46.3)	31/50 (62.0)	0.058	63/87 (72.4)	34/51 (66.7)	0.476
Myasthenic crisis, *n* (%)	5 (3.6)	2 (3.8)	1.000^*^	10 (11.0)	4 (7.5)	0.501
Initial oral GC dose, mg/day, median (IQR)	45 [25, 60]	55 [25, 60]	0.713^†^	60 [40, 60]	60 [45, 60]	0.272^†^
Maximal oral GC dose, mg/day, median (IQR)	60 [30, 60]	60 [35, 60]	0.201^†^	60 [50, 60]	60 [60, 60]	0.105^†^
Maintain oral GC dose, mg/day, median (IQR)	5 [5, 10]	10 [5, 15]	**0.004** ^**†**^	10 [5, 10]	10 [5, 15]	0.173^†^
Pyridostigmine dose, mg/day, median (IQR)	180 [90, 180]	180 [60, 240]	0.750	180 [120, 210]	180 [105, 240]	0.921
IVMP, *n* (%)	42 (30.2)	19 (35.8)	0.454	39 (42.9)	20 (39.6)	0.547
Thymectomy, *n* (%)	24 (17.3)	13 (24.5)	0.254	29 (31.9)	21 (39.6)	0.346

GC, glucocorticoid; MGFA, Myasthenia Gravis Foundation of America; MM, Minimum manifestation; IQR, interquartile range. ^*^Fisher’s exact test. ^†^Mann–Whitney *U*. Values in bold indicate a statistically significant difference between the two groups.

### 3.4 Comparative analysis of early and delayed combination-therapy groups

The Kaplan–Meier curve shows that MM was achieved earlier and more frequently in the early combination-therapy group than in the delayed combination-therapy group (log-rank test, *p* = 0.0299). Univariate Cox regression analysis revealed that the HR and 95% CI were 1.56 and 1.03–2.37, respectively (*p* = 0.036) (Figure 2C).

### 3.5 Comparative analysis of mono-GC and combination-treatment groups

The Kaplan–Meier curve shows that the early mono-GC group reached MM earlier and more frequently than the delayed combination-therapy group (log rank test, *p* = 0.0013). Univariate Cox regression analysis revealed that the HR and 95% CI were 1.83 and 1.24–2.70, respectively (*p* = 0.002) (Figure 2D).

In addition, the Kaplan–Meier curve showed that the early mono-GC group and the early combination-therapy group did not significantly differ in their achievement of treatment goals (log-rank test, *p* = 0.1661; HR, 1.23; *p* = 0.182). The same was true in the delayed mono-GC group and the delayed combination-therapy group (log-rank test, *p* = 0.6269; HR, 0.89; *p* = 0.634) (Figures 2E,F).

### 3.6 Factors associated with response to mono-GC

We evaluated the factors associated with achieving MM status or better in the mono-GC group by using the Cox proportional hazards model to explore predictors affecting prognosis associated with mono-GC (Table 6). In univariate Cox regression analysis, sex, early mono-GC, EOMG, LOMG, OMG, and MGFA I were associated with achieving MM status or better. The inclusion of these variables in multivariate Cox regression analysis showed that early mono-GC (HR: 1.50, *p* = 0.043), EOMG (HR: 1.74, *p* = 0.034), and OMG (HR: 1.90, *p* = 0.007) were positive predictors of MM status or better.

**Table 6:** Variables associated with MM status in mono-GC treatment predicted by Cox proportional hazard model.

variables	Univariate		Multivariate	
	HR (95% CI)	*P-*value	HR (95% CI)	*P-*value
Female (Ref., male)	1.41 (1.00–1.98)	**0.051**	1.26 (0.89–1.77)	0.192
Early mono-GC group (<6 m *VS* ≥ 6 m)	1.66 (1.12–2.45)	**0.011**	1.50 (1.01–2.23)	**0.043**
EOMG (Ref., LOMG)	2.04 (1.40–2.96)	**<0.001**	1.74 (1.04–2.89)	**0.034**
LOMG (Ref., EOMG)	0.57 (0.36–0.92)	**0.021**	0.91 (0.48–1.73)	0.769
OMG (Ref., GMG)	2.14 (1.50–3.07)	**<0.001**	1.90 (1.19–3.04)	**0.007**
MGFA classI(Ref., classII-III)	1.86 (1.19–2.92)	**0.007**	1.02 (0.56–1.84)	0.956
AChR-Ab positive	0.89 (0.57–1.30)	0.467	--	--
MuSK-Ab positive	0.56 (0.18–1.77)	0.325	--	--
SNMG	1.24 (0.78–1.95)	0.363	--	--
Thymectomy	0.69 (0.44–1.08)	0.103	--	--

GC, glucocorticoid; MM, Minimum manifestation; EOMG, early-onset myasthenia gravis; LOMG, late-onset MG; OMG, ocular myasthenia gravis; MGFA, Myasthenia Gravis Foundation of America; AChR-Ab, acetylcholine receptor antibody; MuSK-Ab, muscle-specific tyrosine kinase antibody; SNMG, seronegative myasthenia gravis; HR, hazard ratios; m, months; HR, Hazard ratio; CI, confidence interval. Values in bold indicate a statistically significant difference between the two groups.

### 3.7 Comparison between early and delayed therapy groups across different MG subgroups

In EOMG, OMG, AChR-MG and Titin-MG, the MM status was reached earlier in the early immunotherapy group compared with the delayed immunotherapy group (*p* < 0.05). In the EOMG, AChR-MG and Titin-MG subgroups, there was a difference in clinical severity between the early and delayed immunotherapy groups for the most severe form of the disease (*p* < 0.05), and early immunotherapy groups reduced the maximum oral GC dose in MG patients (*p* < 0.05). In the GMG subgroup, the early immunotherapy group achieved MM status or better more often in the course of MG (*p* = 0.022). In the LOMG subgroup, recurrence occurred less frequently in the early immunotherapy group (*p* = 0.023). In the EOMG, LOMG, GMG, and AChR-MG subgroups, the maintenance oral GC dose in the early immunotherapy group was significantly lower than that in the delayed immunotherapy group (*p* < 0.05) (Supplementary Table S1). In AChR-MG, the early mono-GC group reached MM status earlier than the delayed mono-GC group (*p* = 0.043). In the EOMG, OMG, and AChR-MG subgroups, the maintenance oral GC doses in the early mono-GC group were significantly lower than those in the delayed mono-GC group (5 [5, 10]mg vs. 10 [5, 15]mg, *p* < 0.05) (Supplementary Table S2). We included a total of six patients with MuSK-MG, of whom only two (33.3%) achieved treatment goals for MM status or better. Meanwhile, 203 (71.0%) patients with AChR-MG achieved MM status over the entire course of the disease. However, no statistically significant differences were found between the two groups in achieving treatment goals and oral GC doses. We counted information on 38 seronegative patients, of whom 13 (34.2%) were treated with immunosuppressants in addition to GC, and the remaining 25 (65.8%) were treated with GC alone. Compared with AChR-MG, SNMG experienced less recurrence (58.5% vs. 36.1%, *p* = 0.011) and less thymectomy (*p* = 0.043), and AChR-MG and SNMG did not show a statistically significant difference in achieving treatment target MM or better status (Table 7).

**Table 7:** Comparison between AChR-MG, MuSK-MG and SNMG.

	AChR-MG (*N* = 286)	MuSK-MG (*N* = 6)	SNMG (*N* = 38)	*P_1_-*value	*P_2_-*value
MGFA classification at onset				**0.035** ^*^	0.748
I, *n* (%)	220	2	31		
II, *n* (%)	60	4	6		
III, *n* (%)	6	0	1		
MGFA classification at maximal worsening				**0.016** ^*^	0.525^*^
I, *n* (%)	135	0	22		
II, *n* (%)	55	1	9		
III, *n* (%)	54	4	5		
IV, *n* (%)	22	0	1		
V, *n* (%)	20	1	1		
MM or better status, throughout the course, *n* (%)	203 (71.0)	2 (33.3)	32 (84.2)	0.067^*^	0.086
MM or better status, at last follow up, *n* (%)	158 (55.2)	2 (33.3)	25 (65.8)	0.415^*^	0.218
Relapse, n/N (%)	161/275 (58.5)	2 (33.3)	13/36 (36.1)	0.242^*^	**0.011**
Myasthenic crisis, *n* (%)	19 (6.6)	1 (16.7)	1 (2.6)	0.349^*^	0.488^*^
Maximal oral GC dose, mg/day, median (IQR)	60 [45, 60]	60 [50, 70]	60 [48.75, 60]	0.254^†^	0.290^†^
Maintain oral GC dose, mg/day, median (IQR)	10 [5, 20]	10 [5, 20]	5 [5, 10]	0.097^†^	0.476^†^
Thymectomy, *n* (%)	82 (28.7)	0	5 (13.2)	--	**0.043**

MGFA, Myasthenia Gravis Foundation of America; AChR-MG, Acetylcholine receptor antibody positive Myasthenia Gravis; MuSK-MG, Muscle-specific tyrosine kinase antibody positive Myasthenia Gravis; SNMG, serologically negative MG; MM, Minimum manifestation; GC, glucocorticoid; IQR, interquartile range. ^*^Fisher’s exact test. ^†^Mann–Whitney *U*. *P*_1_-value: Comparison between AChR-MG and MuSK-MG. *P*_2_-value: Comparison between AChR-MG and SNMG. Values in bold indicate a statistically significant difference between the two groups.

## 4 Discussion

In this study, we retrospectively analyzed the clinical data of 336 patients with MG and found that early administration of GC could achieve MM earlier and more frequently and could reduce the maximum and maintenance oral GC doses. Additionally, early mono-GC, EOMG, and OMG were positive predictors of treatment goals. In EOMG, OMG, and AChR-MG, the maintenance oral GC doses in the early mono-GC group were significantly lower than those in the delayed mono-GC group.

In this study, the cervical, limb, and bulbar muscles were more involved in the delayed GC group during MG than the early GC group. First, the etiology and clinical manifestations of MG differ significantly between patients. Some patients may be placed in the early GC group when systemic symptoms appear. Other patients may not receive appropriate treatment for a relatively long time after the onset of systemic symptoms, resulting in disease progression and delayed involvement of the cervical, limb, and bulbar muscles. In addition, MG is a progressive disease whose rate of progression and extent of involvement may vary between patients (1, 22). Some patients may initially be affected by constitutional symptoms, progressing slowly, resulting in delayed cervical, limb, and bulbar muscle involvement. Other patients may have early cervical, limb, and bulbar muscle involvement. Therefore, our findings do not show that patients in the delayed GC group had more severe symptoms of muscle weakness than those in the early GC group, which affected treatment outcomes.

In this study, we analyzed why patients delay GC treatment. Firstly, GC is a potent drug, and long-term use may lead to a series of side effects (6, 13), such as susceptibility to infection, high blood pressure, osteoporosis, etc. Consequently, some patients may be reluctant to take GC because they are concerned about these side effects. Second, some patients may not know enough about the condition and treatment of MG, leading to doubts about the safety and efficacy of GC. Finally, in some patients, symptoms of MG may be relatively mild and can be effectively controlled with other treatments, such as cholinesterase inhibitors. In this case, the doctor will recommend avoiding using GC and regularly monitoring the condition’s progress.

Due to the difficulty of achieving a CSR status, international consensus guidelines recommend defining the actual therapeutic goal of MG as MM status or better (23). In this study, the early immunotherapy group achieved MM earlier and more frequently and needed reduced maximum and maintenance oral GC doses compared with doses in the delayed immunotherapy group. MG is a classic example of antibody-mediated autoimmune disease, and GC and nonsteroidal immunosuppressants are first-line agents in MG therapy. Early immunosuppressive therapy can suppress disease activity earlier, reduce the production of pathogenic antibodies, prevent disease progression, and induce remission. At the same time, other immunosuppressants can act as steroid-sparing agents to reduce the dose of GC to some extent (12, 24). The mechanism through which early GC treatment may lead to higher levels of MM is not fully understood. However, there is a hypothesis that a longer autoimmune attack at the onset of the disease may lead to disruption and structural alteration of autoantigens at the neuromuscular junction. This prolonged exposure may also lead to subsequent exposure to new autoantigens, enhancing the autoimmune attack (25). Early immune intervention can help reduce further tissue destruction and subsequent long-term immune stimulation.

GC comes with a number of potential side effects while treating MG. Long-term side effects of GC include weight gain, other Cushing-like features, easy bruising, cataracts, glaucoma, diabetes mellitus, hypertension, dyslipidemia, osteoporosis, and, rarely, avascular necrosis (6, 13). Side effects of prednisone treatment occurred in 61.0% of patients in this study, with weight gain (37.6%) and Cushing-like appearance (26.7%) being common, followed by complications such as diabetes, hypertension, and osteoporosis. In a long-term retrospective study conducted in the 1980s-90s (5, 6, 26, 27), at least one adverse effect was observed in 41.3–66.7% of MG patients treated with GC, the most common including Cushing-like appearance and weight gain, among others. This is basically consistent with our findings. These adverse effects are related to the dose and duration of GC, and are usually relieved in most patients with dose reduction or alternate-day regimens.

GC are typically used in those patients with MG whose symptoms are not well controlled by cholinesterase inhibitors. Applying GC too late may reduce its efficacy and adversely affect prognosis. In our study, The early mono-GC group achieved treatment targets earlier and more frequently and needed reduced maintenance oral GC doses. Several retrospective studies have shown that early and long-term remission can be achieved with GC treatment in the early course of MG, with 70–80% of MG patients who were treated with GC significantly improving or showing complete symptom remission (5, 6, 27, 28). In our study, 79.1% of patients with MG who were treated with GC achieved MM status or better, and MG symptoms improved significantly.

Recently, two Japanese studies (29, 30) demonstrated that early fast-acting therapy [active use of fast-acting therapies in the early stages, such as plasma exchange, intravenous immunoglobulin, intravenous high-dose methylprednisolone (IVMP)] in patients with GMG within 6 months of initial treatment could achieve the MM5mg target earlier and more, with IVMP being the most effective. IVMP within 3 months of initial treatment in patients with OMG achieved the target of MM5mg earlier and more often than after 3 months and reduced the total dose of oral glucocorticoids. These two studies study the feasibility of routinely administering IVMP therapy to patients with newly diagnosed MG followed by rapid reduction of hormone accumulation while keeping pace with other immunosuppressants for medium to long-term sequential therapy. In addition, a study from Sweden (31) showed that in patients with new-onset systemic MG, patients treated with rituximab earlier met the primary endpoint in a higher proportion compared with placebo. There is a greater interest in early invasive treatments with the rise of the emerging concept of “hit hard and early.” However, further research is needed to address the balance between the long-term benefits and risks of this treatment.

Response to GC therapy in MG patients can be classified as either good or poor. Approximately 5–20% of patients with MG do not respond well to treatment after several weeks or 3 months of high-dose GC therapy (6, 26, 27). High-dose GC therapy in these patients does not increase efficacy but may increase the incidence of adverse effects, and nonsteroidal immunosuppressants should be considered as early as possible (32). This treatment is usually used before or at the beginning of GC reduction. In this study, the early combination-therapy group achieved treatment goals earlier and more frequently than the delayed combination-therapy group. Early combinations of prednisone and other immunosuppressants enable early achievement of MG therapeutic goals (32). In two recent studies, early administration of GC in combination with other calcineurin inhibitors also resulted in earlier and more frequent achievement of MM (29, 30). In our study, we found that the early mono-GC group could achieve treatment goals earlier and more frequently than the delayed combination-therapy group. This may indicate a good efficacy of early mono-GC in patients with mild-to-moderate MG.

In this study, it was found that the early mono-GC group reached MM status earlier and more often than the delayed combination treatment group. Firstly, we emphasized the importance of time. This emphasis on time is because steroids have a potent anti-inflammatory effect that can quickly reduce the symptoms and inflammatory response of the disease, allowing patients to feel significant improvement early in the course of treatment. Early steroid therapy provides more rapid control, stabilization, and reduced risk of progression. In contrast, other immunosuppressants have a slower onset of action, and delayed treatment may take longer to reach MM status (3–5). Second, there may be differences in response to treatment in different patients. Some patients respond well to steroid monotherapy and not well to combination therapy with immunosuppressants. These differences in response to treatment may be related to the patient’s immune system status, the severity of the condition, and individual patient differences (24, 33). Patients who are effective in controlling symptoms with steroids alone may indicate that their disease is milder and that they are more likely to achieve their goal of achieving MM status. Finally, combination immunosuppressive therapy may increase the side effects and risks to which patients are exposed. Immunosuppressants, when used, may lead to decreased immune function, increasing the risk of infections and other adverse effects.

Multivariate Cox regression analysis showed that early mono-GC, EOMG, and OMG were a positive predictor of MM status or better. EOMG seems to be more favorable for achieving MM. In our study, we included patients with EOMG aged 1–18 years of age. MG in children and adolescents in China is mainly ophthalmo-type, which has a higher spontaneous remission rate (34, 35). Moreover, patients with EOMG have few intercurrent diseases and can use steroids and other drugs in sufficient amounts, whereas those with LOMG may be limited in the use of drugs when taking steroids or other immunosuppressants due to concurrent diseases (such as diabetes, abnormal liver and kidney function, etc.), which affects the treatment effect. Finally, some studies have reported no statistically significant difference between EOMG and LOMG in relation to MGFA-PIS grading (36, 37). A series of retrospective studies have demonstrated that early administration of GC reduces the potential rate of generalization and exacerbation of symptoms in patients with OMG, contributes to early improvement of symptoms in OMG, and improves quality of life (8–10).

Compared with AChR-MG, MuSK-MG does not respond well to cholinesterase inhibitors. GC is the most effective drug for the treatment of MuSK-MG, but this form of the disease usually requires high doses of GC. In our study, AChR-MG and MuSK-MG patients did not show statistically significant differences in achieving therapeutic goals and oral GC doses; however, MuSK-MG was significantly less likely to reach therapeutic goals than AChR-MG. MuSK-MG responds well to high doses of steroids (38, 39). Our study did not show a need for higher doses of oral GC in MuSK-MG, which this may be due to the small number of patients included. Most patients with MuSK-MG may require further immunotherapy because a rebound in muscle weakness after GC reduction or discontinuation is common. The diagnosis of SNMG is more challenging due to the lack of specific autoantibodies, and its treatment strategy may differ from AChR-MG and MuSK-MG. There is still some controversy about the therapeutic effect of GC in patients with SNMG. In this study, 65.8% of patients with MG were observed to achieve MM or better, which is broadly consistent with the results reported in Greece and Korea that 35.7 to 61.5% of patients with SNMG achieved MM or better (40, 41), and we observed that SNMG treated with GC reduced recurrence rates compared with AChR-MG. Therefore, we recommend that patients with SNMG should also take GC as soon as possible. Since there are no clear specific autoantibodies in patients with SNMG, individualized treatment strategies need to be developed according to the situation. In addition to GC, other immunomodulatory drugs (e.g., immunoglobulin, tacrolimus, etc.) have also been used in the treatment of patients with SNMG, but their effectiveness still needs to be studied. SNMG is usually milder and less thymic abnormalities, and thymectomy is recommended in adults with thymoma MG and AChR-Ab-positive systemic MG in our country (34, 42). There are no studies evaluating thymectomy in SNMG. 13.2% of patients in our study underwent thymectomy, and more data are needed to complete the study.

This study has several limitations. First, the retrospective study design may introduce selection bias and lacking clinical parameters. Second, the doses and types of immunosuppressants were not compared. Third, due to the small number of patients included in the MuSK-MG subgroup, systematic comparisons with the AChR-MG subgroup were not possible. Fourth, due to various reasons, such as technical limitations, laboratory resources, etc., we have not been able to perform a complete MG4 test for all patients, and further research may include a comprehensive MG4 test to more fully assess the patient’s antibody status. Finally, the number of patients treated with mono-GC in our study was limited, and the sample size should be expanded for prospective clinical studies.

In conclusion, patients with MG who receive early GC treatment can achieve treatment goals earlier and more frequently. Early GC treatment in these patients can also reduce the maintenance oral GC dose, inhibit MG disease activity earlier, and lead to a good prognosis.

## Data availability statement

The original contributions presented in the study are included in the article/Supplementary material, further inquiries can be directed to the corresponding authors.

## Ethics statement

The studies involving humans were approved by Department of Neuroimmunology, Henan Institute of Medical and Pharmaceutical Sciences, Zhengzhou University, Zhengzhou, China. The studies were conducted in accordance with the local legislation and institutional requirements. Written informed consent for participation was not required from the participants or the participants’ legal guardians/next of kin in accordance with the national legislation and institutional requirements.

## Author contributions

LZ: Conceptualization, Writing – original draft. XuZ: Writing – review & editing. WL: Writing – review & editing, Methodology. JW: Methodology, Writing – review & editing. HS: Methodology, Writing – review & editing. SC: Methodology, Writing – review & editing. XiZ: Investigation, Writing – review & editing. YW: Investigation, Writing – review & editing. XY: Investigation, Writing – review & editing. GH: Investigation, Writing – review & editing. ZS: Investigation, Writing – review & editing. YGZ: Writing – review & editing. JZ: Writing – review & editing. HF: Resources, Writing – review & editing. YKZ: Resources, Writing – review & editing. QZ: Resources, Writing – review & editing. XC: Resources, Writing – review & editing. JL: Writing – review & editing. JY: Resources, Writing – review & editing. FG: Conceptualization, Funding acquisition, Writing – review & editing.
