# Supplementary material for: Effectiveness of early glucocorticoids in myasthenia gravis: a retrospective cohort study
Source: Front Neurol. 2023 Dec 19;14:1259484. doi: 10.3389/fneur.2023.1259484 (PMC10770254; doi:10.3389/fneur.2023.1259484)
Supplement: Supplementary file 1 [file Table_1.docx]

**Supplementary Table 1** Comparison between Early and Delayed immunotherapy groups in different MG subgroups

|  | EOMG | | | LOMG | | | TAMG | | | OMG | | | GMG | | | AChR-MG | | | MuSK-MG | | | SNMG | | |
| --- | --- | --- | --- | --- | --- | --- | --- | --- | --- | --- | --- | --- | --- | --- | --- | --- | --- | --- | --- | --- | --- | --- | --- | --- |
|  | Early immnotherapy group (N=135) | Delayed immnotherapy group (N=56) | P value | Early immnotherapy group (N=50) | Delayed immnotherapy group (N=23) | P value | Early immnotherapy group (N=45) | Delayed immnotherapy group (N=27) | P value | Early immnotherapy group (N=123) | Delayed immnotherapy group (N=38) | P value | Early immnotherapy group (N=107) | Delayed immnotherapy group (N=68) | P value | Early immnotherapy group (N=192) | Delayed immnotherapy group (N=94) | P value | Early immnotherapy group (N=4) | Delayed immnotherapy group (N=2) | P value | Early immnotherapy group (N=30) | Delayed immnotherapy group (N=8) | P value |
| MGFA classifification at onset |  |  | 0.584^*^ |  |  | 0.710^*^ |  |  | 0.195^*^ |  |  | -- |  |  | 0.331^*^ |  |  | 0.424^*^ |  |  | 1.000^*^ |  |  | 1.000^*^ |
| I, n (%) | 109 (80.7) | 43 (76.8) |  | 37 (74.0) | 18 (78.3) |  | 32 (71.1) | 19 (70.4) |  | 123 | 38 |  | 55 (51.4) | 42 (61.8) |  | 151 (78.6) | 69 (73.4) |  | 1 (25.0) | 1 (50.0) |  | 23 (76.7) | 1 |  |
| II, n (%) | 25 (18.5) | 12 (21.4) |  | 10 (20.0) | 5 (21.7) |  | 13 (28.9) | 6 (22.2) |  | 0 | 0 |  | 48 (44.9) | 23 (33.8) |  | 38 (19.8) | 22 (23.4) |  | 3 (75.0) | 1 (50.0) |  | 6 (20.0) | 0 |  |
| III, n (%) | 1 (0.7) | 1 (1.8) |  | 3 (6.0) | 0 |  | 0 | 2 (7.4) |  | 0 | 0 |  | 4 (3.7) | 3 (4.4) |  | 3 (1.6) | 3 (3.2) |  | 0 | 0 |  | 1 (3.3) | 0 |  |
| MGFA classifification at maximal worsening |  |  | **0.008^*^** |  |  | 0.715^*^ |  |  | 0.289^*^ |  |  | -- |  |  | 0.545 |  |  | **0.010** |  |  | 1.000^*^ |  |  | 0.772^*^ |
| I, n (%) | 87 (64.4) | 23 (41.1) |  | 22 (44.0) | 7 (30.4) |  | 14 (31.1) | 8 (29.6) |  | 123 | 38 |  | 0 | 0 |  | 104 (54.2) | 31 (33.0) |  | 0 | 0 |  | 16 (53.3) | 6 (75.0) |  |
| II, n (%) | 20 (14.8) | 11 (19.6) |  | 16 (32.0) | 8 (34.8) |  | 5 (11.1) | 5 (18.5) |  | 0 | 0 |  | 41 (38.3) | 24 (35.3) |  | 33 (17.2) | 22 (23.4) |  | 1 (25.0) | 0 |  | 7 (23.3) | 2 (25.0) |  |
| III, n (%) | 20 (14.8) | 12 (21.4) |  | 9 (18.0) | 6 (26.1) |  | 8 (17.8) | 9 (33.3) |  | 0 | 0 |  | 37 (34.6) | 27 (39.7) |  | 29 (15.1) | 25 (26.6) |  | 2 (50.0) | 2 |  | 5 (16.7) | 0 |  |
| Ⅳ, n (%) | 3 (2.2) | 7 (12.5) |  | 2 (4.0) | 1 (4.3) |  | 8 (17.8) | 3 (11.1) |  | 0 | 0 |  | 13 (12.1) | 11 (16.2) |  | 12 (6.3) | 10 (10.6) |  | 0 | 0 |  | 1 (3.3) | 0 |  |
| V, n (%) | 5 (3.7) | 3 (5.4) |  | 1 (2.0) | 1 (4.3) |  | 10 (22.2) | 2 (7.4) |  | 0 | 0 |  | 16 (15.0) | 6 (8.8) |  | 14 (7.3) | 6 (6.4) |  | 1 (25.0) | 0 |  | 1 (3.3) | 0 |  |
| MM or better status, throughout the course, n(%) | 115 (85.2) | 42 (75.0) | 0.094 | 30 (60.0) | 11 (47.8) | 0.330 | 31 (68.9) | 14 (51.9) | 0.148 | 101 (82.1) | 31 (81.6) | 0.940 | 75 (70.1) | 36 (52.9) | **0.022** | 143 (74.5) | 60 (63.8) | 0.062 | 2 (50.0) | 0 | 0.467^*^ | 26 (86.7) | 6 (75.0) | 0.587^*^ |
| MM or better status, at last follow up, n(%) | 94 (69.6) | 33 (58.9) | 0.154 | 22 (44.0) | 7 (30.4) | 0.271 | 22 (48.9) | 11 (40.7) | 0.502 | 84 (68.3) | 26 (68.4) | 0.988 | 54 (50.5) | 25 (36.8) | 0.076 | 113 (58.9) | 45 (47.9) | 0.079 | 2 (50.0) | 0 | 0.467^*^ | 20 (66.7) | 4 (50.0) | 0.433^*^ |
| Relapse, n /N (%） | 79/132 (59.8) | 37/53 (69.8) | 0.205 | 13/46 (28.3) | 12/21 (57.1) | **0.023** | 33/44 (75.0) | 16/25 (64.0) | 0.333 | 64/117 (54.7) | 22/36 (61.1) | 0.498 | 60/104 (57.7) | 43/65 (66.2) | 0.273 | 104/186 (55.9) | 57/89 (64.0) | 0.200 | 2 (50.0) | 0 | 0.467^*^ | 17/28 (60.0) | 6/8 (75.0) | 0.682^*^ |
| Myasthenic crisis, n (%) | 5 (3.7) | 3 (5.4) | 0.694^*^ | 9 (18.0) | 2 (8.7) | 0.484 | 9 (20.0) | 2 (7.4) | 0.191 | 0 | 0 | -- | 15 (14.0) | 6 (8.8) | 0.303 | 13 (6.8) | 6 (6.4) | 0.902 | 1 (25.0) | 0 | 1.000^*^ | 1 (3.3) | 0 | 1.000^*^ |
| Maximal oral GC dose, mg/day, median (IQR) | 40 [30, 60] | 60 [30, 60] | **0.027^†^** | 60 [60, 60] | 60 [60, 60] | 0.384^†^ | 60 [60, 60] | 60 [60, 60] | 0.440^†^ | 40 [25, 60] | 60 [30, 60] | 0.112^†^ | 60 [60, 60] | 60 [60, 60] | 0.724^†^ | 60 [30, 60] | 60 [45, 60] | **0.011^†^** | -- | -- | -- | 60 [45, 60] | 60 [52.5, 60] | 0.879 |
| Maintain oral GC dose, mg/day, median (IQR) | 5 [5, 10] | 10 [5, 10] | **0.036^†^** | 5 [5, 10] | 10 [5, 20] | **0.003^†^** | 10 [5, 12.5] | 10 [5, 10] | 0.584^†^ | 5 [5, 10] | 10 [5, 11.25] | **0.017^†^** | 10 [5, 10] | 10 [5, 15] | 0.094^†^ | 5 [5, 10] | 10 [5, 15] | **0.001^†^** | -- | -- | -- | 5 [5, 10] | 5 [5, 17.5] | 0.692 |
| Thymectomy, n (%) | 6 (4.4) | 7 (12.5) | 0.059^*^ | 2 | 0 | 1.000^*^ | 45 | 27 | -- | 16 (13.0) | 10 (26.3) | 0.051^*^ | 37 (34.6) | 24 (35.3) | 0.923^*^ | 50 (26.0) | 32 (34.0) | 0.160^*^ | 0 | 0 | -- | 3 (10.0) | 2 (25.0) | 0.279^*^ |

*MG,* myasthenia gravis; *EOMG,* Early-onset MG; *LOMG,* Late-onset MG; *TAMG,* thymoma MG; *OMG,* ocular MG; *GMG,* generalized MG; *AChR-MG,* Acetylcholine receptor antibody positive myasthenia Gravis; *MuSK-MG,* Muscle-specific tyrosine kinase antibody positive MG; *SNMG,* serologically negative MG; *MGFA,* Myasthenia Gravis Foundation of America; *MM,* Minimum manifestation; *GC,* glucocorticoid; *IQR,* interquartile range.

^*^Fisher’s exact test

^†^Mann-Whitney U
