# Supplementary material for: Effectiveness of early glucocorticoids in myasthenia gravis: a retrospective cohort study
Source: Front Neurol. 2023 Dec 19;14:1259484. doi: 10.3389/fneur.2023.1259484 (PMC10770254; doi:10.3389/fneur.2023.1259484)
Supplement: Supplementary file 2 [file Table_2.docx]

**Supplementary Table 2** Comparison between Early and Delayed mono-GC groups in different MG subgroups

|  | EOMG | | | LOMG | | | TAMG | | | OMG | | | GMG | | | AChR-MG | | | MuSK-MG | | | SNMG | | |
| --- | --- | --- | --- | --- | --- | --- | --- | --- | --- | --- | --- | --- | --- | --- | --- | --- | --- | --- | --- | --- | --- | --- | --- | --- |
|  | Early mono-GC group (N=93) | Delayed mono-GC group (N=31) | P value | Early mono-GC group (N=27) | Delayed mono-GC group (N=10) | P value | Early mono-GC group (N=19) | Delayed mono-GC group (N=12) | P value | Early mono-GC group (N=90) | Delayed mono-GC group (N=28) | P value | Early mono-GC group (N=49) | Delayed mono-GC group (N=25) | P value | Early mono-GC group (N=112) | Delayed mono-GC group (N=45) | P value | Early mono-GC group (N=2) | Delayed mono-GC group (N=2) | P value | Early mono-GC group (N=21) | Delayed mono-GC group (N=4) | P value |
| MGFA classifification at onset |  |  | 1.000^*^ |  |  | 0.478^*^ |  |  | 0.319^*^ |  |  | -- |  |  | 0.868^*^ |  |  | 0.468^*^ |  |  | -- |  |  | 0.617^*^ |
| I, n (%) | 78 (83.9) | 27 (87.1) |  | 20 (74.1) | 7 (70.0) |  | 14 (73.7) | 7 (58.3) |  | 90 | 28 |  | 22 (44.9) | 13 (52.0) |  | 94 (83.9) | 34 (75.6) |  | 0 | 1 |  | 15 (71.4) | 4 |  |
| II, n (%) | 14 (15.1) | 4 (12.9) |  | 4 (14.8) | 3 (30.0) |  | 5 (26.3) | 3 (25.0) |  | 0 | 0 |  | 23 (46.9) | 10 (40.0) |  | 15 (13.4) | 9 (20.0) |  | 2 | 1 |  | 5 (23.8) | 0 |  |
| III, n (%) | 1 (1.1) | 0 |  | 3 (11.1) | 0 |  | 0 | 2 (16.7) |  | 0 | 0 |  | 4 (8.2) | 2 (8.0) |  | 3 (2.7) | 2 (4.4) |  | 0 | 0 |  | 1 (4.8) | 0 |  |
| MGFA classifification at maximal worsening |  |  | 0.058^*^ |  |  | 0.627^*^ |  |  | 0.477^*^ |  |  | -- |  |  | 0.485^*^ |  |  | 0.222^*^ |  |  | -- |  |  | 0.238^*^ |
| I, n (%) | 64 (68.8) | 20 (64.5) |  | 15 (55.6) | 4 (40.0) |  | 11 (57.9) | 4 (33.3) |  | 90 | 28 |  | 0 | 0 |  | 77 (68.8) | 23 (51.1) |  | 0 | 0 |  | 10 (47.6) | 4 |  |
| II, n (%) | 15 (16.1) | 3 (9.7) |  | 4 (14.8) | 3 (30.0) |  | 0 | 1 (8.3) |  | 0 | 0 |  | 19 (38.8) | 7 (28.0) |  | 12 (10.7) | 7 (15.6) |  | 0 | 0 |  | 7 (33.3) | 0 |  |
| III, n (%) | 13 (14.0) | 5 (16.1) |  | 5 (18.5) | 2 (20.0) |  | 3 (15.8) | 4 (33.3) |  | 0 | 0 |  | 21 (42.9) | 11 (44.0) |  | 14 (12.5) | 9 (20.0) |  | 2 | 2 |  | 4 (19.0) | 0 |  |
| Ⅳ, n (%) | 0 | 3 (9.7) |  | 2 (7.4) | 0 |  | 2 (10.5) | 2 (16.7) |  | 0 | 0 |  | 4 (8.2) | 5 (20.0) |  | 4 (12.5) | 4 (8.9) |  | 0 | 0 |  | 0 | 0 |  |
| V, n (%) | 1 (1.1) | 0 |  | 1 (3.7) | 1 (10.0) |  | 3 (15.8) | 1 (8.3) |  | 0 | 0 |  | 5 (10.2) | 2 (8.0) |  | 5 (4.5) | 2 (4.4) |  | 0 | 0 |  | 0 | 0 |  |
| MM or better status, throughout the course, n(%) | 82 (88.2) | 24 (77.4) | 0.151^*^ | 16 (59.3) | 4 (40.0) | 0.460^*^ | 12 (63.2) | 5 (41.7) | 0.242 | 76 (84.4) | 21 (75.0) | 0.267^*^ | 34 (69.4) | 12 (48.0) | 0.073 | 86 (76.8) | 29 (64.4) | 0.114 | 1 | 0 | -- | 19 (90.5) | 3 (75.0) | 0.422^*^ |
| MM or better status, at last follow up, n(%) | 69 (74.2) | 21 (67.7) | 0.486 | 12 (44.4) | 4 (40.0) | 1.000^*^ | 9 (47.4) | 3 (25.0) | 0.274^*^ | 63 (70.0) | 18 (64.3) | 0.569 | 27 (55.1) | 9 (36.0) | 0.120 | 71 (63.4) | 23 (51.5) | 0.156 | 1 | 0 | -- | 15 (71.4) | 3 (75.0) | 1.000^*^ |
| Relapse, n /N (%） | 46/91 (50.0) | 18/28 (64.3) | 0.202 | 5/25 (20.0) | 4/10 (40.0) | 0.393^*^ | 11/18 (61.1) | 9/12 (75.0) | 0.694^*^ | 40/85 (47.1) | 16/26 (61.5) | 0.196 | 22/49 (44.9) | 15/24 (62.5) | 0.158 | 52/109 (47.7) | 27/42 (64.3) | 0.068 | 0 | 0 | -- | 9/20 (45.0) | 2 | 0.476^*^ |
| Myasthenic crisis, n (%) | 1 | 0 | -- | 1 (3.7) | 1 (10.0) | 0.473^*^ | 3 (15.8) | 1 (8.3) | 1.000^*^ | 0 | 0 | -- | 5 (10.2) | 2 (8.0) | 1.000^*^ | 5 (4.5) | 2 (4.4) | 1.000^*^ | 0 | 0 | -- | 0 | 0 | -- |
| Maximal oral GC dose, mg/day, median (IQR) | 35 [25, 60] | 40 [22.5, 60] | 0.532^†^ | 60 [60, 60] | 60 [60, 62.5] | 0.294^†^ | 60 [60, 60] | 60 [48.75, 60] | 0.343^†^ | 35 [25, 60] | 60 [26.25, 60] | 0.232^†^ | 60 [60, 60] | 60 [47.5, 60] | 0.606^†^ | 42.5 [26.25, 60] | 60 [30, 60] | 0.120^†^ | -- | -- | -- | 60 [55, 60] | 60 [31.88, 60] | 0.767^†^ |
| Maintain oral GC dose, mg/day, median (IQR) | 5 [5, 10] | 10 [5, 15] | **0.017^†^** | 5 [5, 10] | 7.5 [5, 12.5] | 0.422^†^ | 10 [5, 10] | 10 [5, 15] | 0.443^†^ | 5 [5, 10] | 7.5 [5, 13.75] | **0.019^†^** | 10 [5, 10] | 10 [5, 15] | 0.222^†^ | 5 [5, 10] | 10 [5, 15] | **0.010^†^** | -- | -- | -- | 5 [5, 10] | 7.5 [5, 17.5] | 0.437^†^ |
| Thymectomy, n (%) | 3 (3.2) | 1 (3.2) | 1.000^*^ | 2 | 0 | **--** | 19 | 12 | -- | 13 (14.4) | 5 (17.9) | 0.764^*^ | 11 (22.4) | 8 (36.0) | 0.214 | 23 (20.5) | 13 (28.9) | 0.260 | 0 | 0 | -- | 1 | 0 | -- |

*MG,* myasthenia gravis; *EOMG,* Early-onset MG; *LOMG,* Late-onset MG; *TAMG,* thymoma MG; *OMG,* ocular MG; *GMG,* generalized MG; *AChR-MG,* Acetylcholine receptor antibody positive myasthenia Gravis; *MuSK-MG,* Muscle-specific tyrosine kinase antibody positive MG; *SNMG,* serologically negative MG; *MGFA,* Myasthenia Gravis Foundation of America; *MM,* Minimum manifestation; *GC,* glucocorticoid; *IQR,* interquartile range.

^*^Fisher’s exact test

^†^Mann-Whitney U
